# Surgical Versus Conservative Management for Carpal Tunnel Syndrome: An Updated Systematic Review of Randomised Trials

**DOI:** 10.3390/brainsci16040399

**Published:** 2026-04-08

**Authors:** Sara Masiero, Pasquale Arcuri, Paolo Boccolari, Elena Zorzi, Alessandro Vio, Tracy Fairplay, Davide Zanin, Fabio Vita, Danilo Donati, Roberto Tedeschi

**Affiliations:** 1 MediClinic, 35020 Pozzonovo, Italy; 2 Fairplay-Arcuri Private Functional Hand Rehabilitation, 40100 Bologna, Italy; 3 Physical Therapy and Rehabilitation Unit, Policlinico di Modena, 41125 Modena, Italy; 4 Azienda ULSS 6 Euganea, U.O.C. Medicina Fisica e Riabilitazione, Ospedale Camposampiero, 35020 Padova, Italy; 5 Independent Researcher and Clinician, inFisio, 47893 Borgo Maggiore, San Marino; 6 Independent Resercher, 10121 Torino, Italy; 7 IRCCS Istituto Ortopedico Rizzoli, 40136 Bologna, Italy; 8 Clinical and Experimental Medicine PhD Program, University of Modena and Reggio Emilia, 41121 Modena, Italy; 9 Independent Researcher, 40100 Bologna, Italy

**Keywords:** carpal tunnel syndrome, surgical versus conservative management, evidence-based rehabilitation, nerve decompression, functional recovery

## Abstract

**Highlights:**

**What are the main findings?**

- Conservative treatments provide faster short-term symptom relief.
- Surgery yields a higher probability of long-term clinical recovery.

**What are the implications of the main findings?**

- Conservative care may be suitable for rapid symptom control.
- Surgery should be considered for durable functional recovery.

**Abstract:**

**Background:** Carpal tunnel syndrome (CTS) is one of the most common entrapment neuropathies. While surgical decompression is widely considered the definitive treatment, conservative options remain clinically relevant, particularly for symptom relief and functional recovery in the short term. **Objectives:** To update the evidence comparing surgical versus non-surgical interventions for CTS, assessing pain, function, and clinical recovery. **Design:** Systematic review of randomised controlled trials (RCTs). **Data Sources and Methods:** Six databases (CENTRAL, MEDLINE, Embase, Cochrane Neuromuscular Register, ClinicalTrials.gov, and WHO ICTRP) were searched for RCTs published between November 2022 and January 2025. Risk of bias was assessed with RoB 2.0 and certainty of evidence with GRADE. Due to clinical heterogeneity, a narrative synthesis was performed. **Results:** Four RCTs (*n* = 1158) were included. Corticosteroid injection and percutaneous electrical nerve stimulation (PENS) appeared to provide faster symptom relief than surgery at short-term follow-up. However, surgery was associated with a higher probability of sustained recovery at 12–18 months (RR 1.36; 95% CI 1.19–1.56). Evidence for PENS was limited to one female-only trial, which restricts generalisability. Certainty of evidence was moderate for long-term outcomes and low for short-term results and safety. **Conclusions:** The available evidence suggests that surgery may offer more durable long-term recovery, whereas corticosteroids and PENS may be useful for short-term symptom relief. These findings should be interpreted with caution given the limited number of trials and the risk of bias in most included studies. Treatment choice should align with patient goals and recovery timelines. Registration: PROSPERO (CRD420250650789).

## 1. Introduction

Carpal tunnel syndrome (CTS) is the most frequently occurring peripheral entrapment neuropathy, affecting between 1% and 5% of the adult population worldwide [1]. The underlying mechanisms and the most effective therapeutic strategies for this condition remain the subject of ongoing scientific debate [1,2]. CTS results from increased pressure and mechanical strain on the median nerve within the carpal tunnel, leading to paraesthesia, nocturnal pain, and, in advanced cases, motor weakness and atrophy of the thenar muscles, with substantial impairments in manual dexterity and quality of life [3,4,5].

The pathophysiology of CTS is multifactorial. Evidence indicates that both raised intracarpal pressure and traction-induced neuropathy are central mechanisms [6]. These changes disrupt venous drainage and promote fluid accumulation, leading progressively to ischaemia, demyelination, axonal degeneration, and fibrosis, ultimately impairing nerve conduction [7].

Typical symptoms include tingling, burning, or numbness in at least two of the three digits innervated by the median nerve—the thumb, index, and middle fingers [8]. In severe or chronic cases, prolonged compression results in thenar eminence muscle atrophy and markedly compromised hand function [9].

Globally, the CTS prevalence varies considerably, with recent meta-analyses reporting rates as high as 7–10% in some regions [9,10]. The condition is more common in women, likely due to hormonal influences and smaller carpal tunnel dimensions [11,12,13,14].

The diagnostic approach to CTS currently relies on a combination of clinical evaluation, provocative manoeuvres, and sensory–motor tests, supported when needed by imaging and electrophysiological studies [1,15,16,17,18,19]. Although ultrasound, MRI, and electrodiagnostic studies are widely used, no single method provides absolute diagnostic accuracy [20]. Among instrumental techniques, high-resolution ultrasound has shown diagnostic accuracy comparable to that of nerve conduction studies [1,6,21], allowing the detection of morphological changes in the median nerve, reduced gliding capacity, and increased cross-sectional area (CSA) [22].

Management strategies for CTS are broadly categorised as conservative or surgical. Conservative treatments include splinting, non-steroidal anti-inflammatory drugs, corticosteroid injections, and targeted physiotherapy, which aims to reduce mechanical stress on the nerve through joint mobilisation, nerve and tendon gliding exercises, and ergonomic education [23,24]. Modifications in workplace ergonomics and patient education have been shown to prevent progression and enhance symptom relief in the hope of reducing the elevated costs incurred by the healthcare system when treating this syndrome [25].

Surgical decompression involves the division of the transverse carpal ligament, performed through either traditional open or minimally invasive endoscopic techniques. The latter are associated with faster recovery and lower postoperative complication rates [26,27]. According to the American Academy of Orthopaedic Surgeons (AAOS) 2024 guidelines, surgery is indicated for patients with persistent or progressive symptoms who are unresponsive to adequate conservative management or in the presence of neurological deficits or electrodiagnostic evidence of severe neuropathy [28]. In acute traumatic CTS, surgery is an urgent intervention that usually ensures prompt relief and substantial functional improvement.

A recent Cochrane review by Lusa et al. (2024) [26] compared surgical and non-surgical interventions, confirming superior short-term symptom relief following surgery yet acknowledging the validity of conservative treatment, particularly in early or mild cases. The appropriate therapeutic decision should therefore be individualised, considering symptom severity, patient preferences, and the risk–benefit ratio. Given the substantial socioeconomic burden of CTS (including high healthcare costs, work absenteeism, and reduced productivity), updating evidence-based recommendations is of particular importance.

The present review was conceived as an update to the Cochrane review by Lusa et al. [26], with a specific focus on recently published randomised controlled trials. Therefore, this review aims to critically evaluate the comparative effectiveness of surgical and conservative treatments for carpal tunnel syndrome by synthesising evidence from randomised controlled trials (RCTs) published between 2023 and 2025. Specifically, it investigates whether surgical decompression of the median nerve provides superior short- and long-term clinical outcomes compared with conservative interventions such as splinting, corticosteroid injections, and physiotherapy.

## 2. Methods

### 2.1. Study Design

This systematic review was designed to compare the effectiveness of surgical and non-surgical interventions for carpal tunnel syndrome (CTS), including exclusively randomised controlled trials (RCTs). The review was conducted in accordance with the PRISMA 2020 statement [27] and registered in PROSPERO (ID: CRD420250650789) on 20 March 2025, after completion of the literature search but before data extraction and analysis. Although registration occurred prior to analysis, it should not be considered fully prospective, and this represents a methodological limitation.

### 2.2. Eligibility Criteria

Only RCTs were considered eligible, irrespective of publication status, language, or outcome domains. When full-text articles were unavailable, the corresponding authors were contacted directly to request access.

### 2.3. Participants

Eligible studies included adults with a clinical or instrumental diagnosis of CTS, regardless of diagnostic criteria, aetiology, comorbidities, sex, or age. For studies including multiple compression neuropathies, only data pertaining specifically to CTS were extracted when separable.

### 2.4. Interventions

Trials comparing any surgical technique (open, mini-open, or endoscopic carpal tunnel release) against any conservative or sham intervention (e.g., manual therapy, splinting, corticosteroid injection, or physical therapy) were included.

### 2.5. Outcomes

No outcome restrictions were applied during study selection. All patient-reported outcome measures (PROMs) reported by the authors were included. The primary outcomes were

- Symptoms and function, preferably measured with the Boston Carpal Tunnel Questionnaire (BCTQ) or other validated instruments;
- Pain intensity, assessed using the Visual Analogue Scale (VAS), Numeric Pain Rating Scale (NPRS), or equivalent tools;
- Health-related quality of life, as measured by validated questionnaires;
- Need for surgery, particularly in participants initially assigned to conservative care or following surgical failure.

Outcomes were categorised as short-term (≤3 months), mid-term (6–9 months), and long-term (≥12 months).

### 2.6. Exclusion Criteria

All studies not fulfilling these criteria were excluded.

### 2.7. Search Strategy

A comprehensive search was conducted across six electronic databases: the Cochrane Central Register of Controlled Trials (CENTRAL), the Cochrane Neuromuscular Specialised Register, MEDLINE (Ovid), Embase (Ovid), ClinicalTrials.gov, and the WHO International Clinical Trials Registry Platform (ICTRP).

Search strategies were developed in accordance with the PRISMA-S [28] and adapted from the previous Cochrane review. The search was intentionally limited to studies published between November 2022 and January 2025 in order to provide an updated synthesis of the most recent evidence following the latest Cochrane [26] review and to avoid duplication of previously synthesised trials. The final search was performed on 30 January 2025. Additionally, the reference lists of all included studies and relevant reviews were hand-searched to identify further eligible RCTs. The complete search strategy is available in the Supplementary Materials.

### 2.8. Study Selection

**Figure 1:**
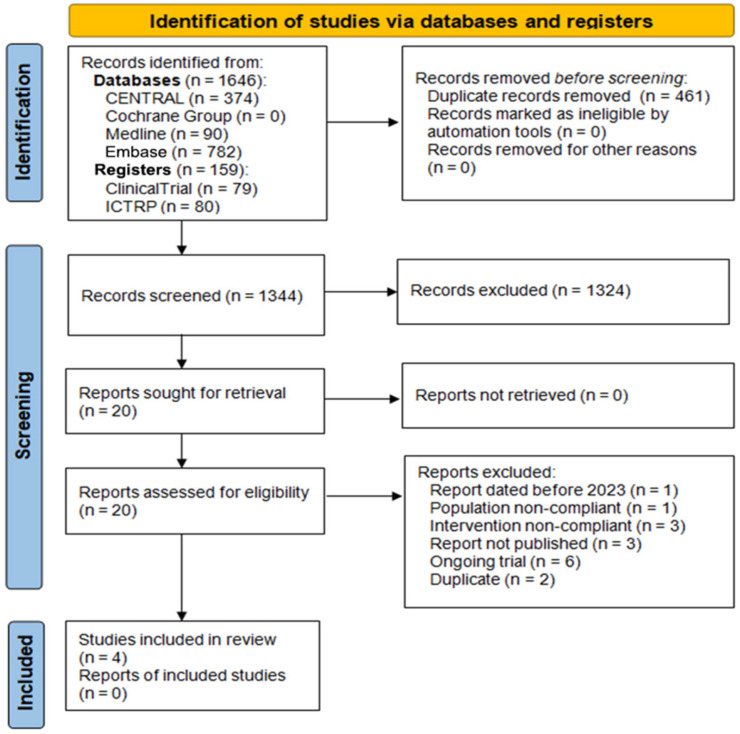
PRISMA 2020 flow diagram of study selection process.

All retrieved records were imported into Rayyan QCRI for title and abstract screening. Duplicates were removed using Deduplicator, an official component of the Systematic Review Accelerator suite [28]. Two reviewers independently screened all records and subsequently evaluated the full texts of potentially relevant studies. Any discrepancies were resolved through discussion or consultation with a third reviewer. Reasons for exclusion were recorded in detail and summarised in a PRISMA 2020 flow diagram (Figure 1) [29]. Reference management was performed with Zotero.

### 2.9. Data Extraction

Data extraction was independently conducted by two reviewers using a pre-piloted standardised form, with arbitration by a third reviewer when disagreements arose. Extracted data included

- General characteristics of the study (authors, year, country, design);
- Demographic and clinical features of participants;
- Details of interventions and comparators;
- Outcome measures and follow-up durations;
- Main findings and authors’ conclusions;
- Funding sources and conflicts of interest.

When both endpoint and change-from-baseline data were available, endpoint data were prioritised. Intention-to-treat (ITT) analyses were preferred over per-protocol or as-treated analyses to minimise attrition bias.

### 2.10. Risk of Bias Assessment

The methodological quality of each included study was independently assessed by two reviewers using the Cochrane Risk of Bias 2.0 (RoB 2) tool [30], with visual summaries generated through RobVis [31]. Five domains were evaluated: randomisation process (D1), deviations from intended interventions (D2), missing outcome data (D3), outcome measurement (D4), and selection of reported results (D5).

The overall risk of bias was classified as “low”, “some concerns”, or “high” following the Cochrane methodology guidelines. Ratings were deemed “high” if any domain was high or if multiple domains were judged as having some concerns. The “some concerns” category was applied when a single domain raised moderate uncertainty.

Additional domain-specific criteria were introduced to increase the precision of appraisal. For D2, studies were rated “high” when crossovers or withdrawals precluded ITT analysis or introduced differential deviations. For D3, attrition exceeding 20% in any group was considered “high risk”, and 10–20% as “some concerns”, in line with commonly adopted methodological conventions for attrition bias and with guidance from the Cochrane Handbook [32]. For D4, when PROMs were self-reported and treatment awareness was likely to influence responses, bias was rated as “high”. For D5, the absence of a published protocol or lack of pre-specified outcomes led to a rating of “some concerns”.

These refinements ensured consistent assessment across the four included RCTs and alignment with the prior Cochrane methodology.

### 2.11. Data Synthesis and Statistical Approach

Given the clinical and methodological heterogeneity among studies—different comparators (e.g., surgery vs. corticosteroid injection, surgery vs. PENS), inconsistent outcome instruments (BCTQ, VAS/NPRS, CTS-6), and variable follow-up durations—a meta-analysis was deemed inappropriate.

A narrative synthesis without meta-analysis (SWiM) was therefore performed, in accordance with the SWiM reporting guidelines [1], the Cochrane Handbook Chapter 12 [32], and the PRISMA 2020 recommendations [33]. Studies were grouped by comparator and follow-up timeframe (short-, mid-, and long-term). For continuous outcomes, effect sizes were reported as mean differences (MDs) with 95% confidence intervals (CIs) and, for dichotomous outcomes, as a risk ratio (RR) or risk difference (RD), supplemented by the number needed to treat (NNT) or number needed to harm (NNH) when applicable.

For scales where lower scores reflected a clinical improvement (VAS, NPRS, BCTQ, QuickDASH), a negative MD favoured surgery. Conversely, for outcomes where higher scores indicated better results (e.g., global recovery, satisfaction), the interpretation was adjusted accordingly without inverting the scale direction.

Heterogeneity was qualitatively appraised considering population characteristics, intervention modalities, comparator types, measurement tools, timing, and risk of bias at the outcome level. Sensitivity analyses were planned to exclude studies with a high risk of bias; however, data limitations restricted their feasibility. Vote counting based solely on *p*-values was explicitly avoided.

### 2.12. Certainty of Evidence

The certainty of the body of evidence was appraised using the GRADE framework and summarised in a Summary of Findings (SoF); see the table in Section 3.5. The assessment considered risk of bias, inconsistency, indirectness, imprecision, and potential publication bias. Downgrading decisions were explicitly justified for each outcome.

### 2.13. Protocol Amendments

The original protocol included a quantitative synthesis (meta-analysis) contingent upon sufficient homogeneity across studies. However, prior to data extraction and analysis, the decision was made to proceed with a narrative synthesis due to substantial heterogeneity in outcome measures, comparators, and follow-up durations across the four eligible RCTs. This methodological adaptation was made independently of the observed results to preserve analytical neutrality.

## 3. Results

### 3.1. Study Selection

The comprehensive search identified 1805 records, of which 1646 originated from bibliographic databases (CENTRAL, *n* = 374; MEDLINE, *n* = 490; Embase, *n* = 782) and 159 from trial registries (ClinicalTrials.gov, *n* = 79; WHO-ICTRP, *n* = 80). After the removal of 461 duplicates, 1344 unique records were screened by title and abstract, and, of these, 1324 were excluded as clearly irrelevant or ineligible, leaving 20 full-text articles for assessment. Sixteen studies were subsequently excluded for the following reasons: non-congruent intervention or comparator (*n* = 3), publication date prior to the pre-specified range (*n* = 1), non-eligible population (*n* = 1), completed but unpublished (*n* = 3), duplicates (*n* = 2), still recruiting (*n* = 3), or not yet recruiting (*n* = 3).

Ultimately, four randomised controlled trials (RCTs) met all inclusion criteria and were retained for qualitative synthesis. The study selection process is illustrated in the PRISMA 2020 flow diagram (Figure 1).

The diagram illustrates the identification, screening, eligibility, and inclusion phases of the systematic review according to the PRISMA 2020 statement.

### 3.2. Characteristics of Included Studies

The four RCTs (Table 1), published between 2023 and 2025, included a total of 1158 adults with clinically or instrumentally confirmed carpal tunnel syndrome (CTS). Sample sizes ranged from 70 to 934 participants.

Mean participant ages were between 39 and 59 years, with a marked female predominance (54–100%). One study specifically recruited only women with unilateral CTS [34]. Symptom duration ranged from approximately 3 months to over 21 months, capturing both subacute and chronic cases.

Three trials compared carpal tunnel release (CTR)—performed via either open or endoscopic techniques—with local corticosteroid injection, using different active agents (e.g., triamcinolone, methylprednisolone). The remaining trial compared endoscopic CTR with ultrasound-guided percutaneous electrical nerve stimulation (PENS), a minimally invasive neuromodulatory intervention. Follow-up durations ranged from 6 weeks to 18 months, allowing short-, mid-, and long-term comparisons of symptom relief and functional recovery. A structured summary of the study characteristics is presented in Table 1.

**Table 1:** Summary of included randomised controlled trials. Summary of characteristics and main findings of the randomised controlled trials (RCTs) included in the review. CTS = Carpal Tunnel Syndrome; CTR = Carpal Tunnel Release; EMG = Electromyography; VAS = Visual Analogue Scale; NPRS = Numeric Pain Rating Scale; BCTQ = Boston Carpal Tunnel Questionnaire; GROC = Global Rating of Change; PENS = Percutaneous Electrical Nerve Stimulation; F = Female; M = Male; RCT = Randomised Controlled Trial.

Study Design	Setting, Participants (*n*) Mean Age (Years) Sex Mean Symptom Duration	Inclusion/Exclusion Criteria	Intervention	Control	Follow-Up	Primary Outcomes Secondary Outcomes	Main Results Conclusions
Shahab et al., 2023 RCT [35]	Department of Orthopaedics, Gomal Medical College, Pakistan 74 (37 surgery, 37 steroids) 39.6 ± 2.86 (steroids), 38.7 ± 3.28 (surgery) 56.8% F (steroids), 54% F (surgery) 9 ± 2.1 mo (steroids), 8 ± 1.8 mo (surgery)	≥16 yrs, clinical + EMG diagnosis; excluded recurrences, fractures, diabetes, pregnancy, other neuropathies	Single injection: 20 mg methylprednisolone + 2 mL xylocaine	Open carpal tunnel release (local anaesthesia)	Baseline, 3, 6, 12 months	Pain (VAS), numbness (Michael-Griffin)	3 mo: improvement in numbness 8% surgery vs. 19% steroids; pain 5% surgery vs. 19% steroids. 12 mo: pain-free 92% both groups; numbness-free 92% surgery vs. 86% steroids. Comparable results at 12 months.
Minoğlu et al., 2023 RCT [34]	Department of Neurosurgery, Atatürk Training and Research Hospital, İzmir, Turkey 80 (36 surgery, 44 steroids) 46.4 ± 9.7 71 F, 9 M 21.8 mo	20–70 yrs, clinical + EMG CTS; excluded diabetes, pregnancy, trauma, other neuropathies, RA, fractures, deformities	Single injection: 6 mg betamethasone (3 + 3 mg)	Open CTR (curvilinear incision, local anaesthesia)	1, 3, 6, 12 mo	Nocturnal paraesthesia (complete disappearance = success) BI-Q symptomatic + functional scales, Hi-Ob, VAS	3 mo: success 59% steroids, 100% surgery; 12 mo: 32% steroids, 100% surgery. Surgery superior; steroids suitable for unilateral mild/moderate CTS.
Palmbergen et al., 2025 (DISTRICTS) RCT [36]	31 hospitals, The Netherlands (neurology clinics) 934 (468 surgery, 466 steroids) 59.0 (51–69) steroids; 58.0 (48–71) surgery 59% F (steroids), 57% F (surgery) 9.0 (5–23 mo) steroids; 8.0 (4–24 mo) surgery	Clinical + EMG/ultrasound CTS; excluded prior surgery/injection < 1 yr, other neuropathies	Corticosteroid injection (protocols varied by centre)	CTR (open or endoscopic, varied by centre)	6 wk, 3, 6, 9, 12, 15, 18 mo	Clinical recovery (CTS-6 < 8 at 18 mo) Time to recovery, QuickDASH, palmar pain, activity limitation, patient satisfaction (Likert items), additional treatments	18 mo: Recovery in 61% surgery vs. 45% steroids (RR 1.36, 95% CI 1.19–1.56). Surgery yields higher long-term recovery probability.
Fernández-de-las-Peñas et al., 2023 RCT [37]	Regional Hospital of Madrid, Spain 70 (35 surgery, 35 PENS) 46 ± 10 (PENS), 47 ± 7 (surgery) 100% F 3.8 ± 0.6 (PENS), 3.7 ± 1.4 (surgery)	Women < 65 yrs; CTS clinical + EMG; excluded bilateral, men, trauma, other neuropathies, pregnancy, systemic disease, fibromyalgia	3 weekly sessions of ultrasound-guided PENS of the median nerve (regional anaesthesia)	Endoscopic CTR	Baseline, 1, 3, 6, 12 mo	Pain (NPRS) Function + symptom severity (BCTQ), GROC	3 mo: pain 2.6 surgery vs. 1.2 PENS; function 1.8 vs. 1.25. 12 mo: pain 1.25 vs. 1.2; function 1.35 vs. 1.2. PENS superior short-term; similar at 12 mo.

### 3.3. Risk of Bias

**Figure 2:**
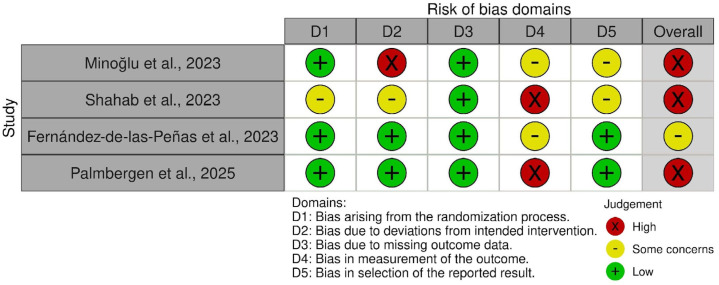
Risk of bias (ROB 2) [34,35,36,37].

Figure 2 presents the risk of bias assessment using the Cochrane RoB 2.0 tool.

Three RCTs were judged at an overall high risk of bias and one study with some bias concerns. The most critical domains were D4 (measurement of outcomes)—given the reliance on subjective patient-reported outcome measures (PROMs) in open-label contexts—and D2 (deviations from intended interventions), especially where non-intention-to-treat analyses or crossovers occurred.

In Minoğlu et al. 2023 [34], randomisation and allocation concealment were clearly described, and follow-up completeness was excellent. However, multiple crossovers from the surgical to the steroid group led to analysis by treatment received rather than by randomised allocation, constituting a high risk for D2. Although blinded assessors were used for follow-up visits (with wrist casting to mask scars), patients were aware of their treatments, potentially influencing PROMs such as VAS and BCTQ—hence, “some concerns” were noted for D4. The absence of a verifiable protocol led to some concerns for D5, and the overall judgement was considered “high risk”.

In Shahab et al., 2023 [35], allocation concealment was not reported, and the study was open-label. The use of self-reported primary outcomes without administering a blind study, which would likely introduce expectation bias, led to a high risk for D4 and overall high risk.

Fernández-de-las-Peñas et al. 2023 [37] (PENS vs. surgery, women only) showed substantial randomisation and allocation concealment, adherence to ITT principles, and minimal attrition. Although participants were unblinded, prior unfamiliarity with either procedure reduced expectation bias, and treatment education was delivered symmetrically. D4 was rated as “some concerns”, while other domains were “low risk”, resulting in an overall judgement of “some concerns”.

The DISTRICTS trial (Palmbergen et al., 2025 [36]), published in *The Lancet*, applied centralised randomisation, ITT analysis, and sensitivity testing with multiple imputation for 10–20% attrition. However, the open-label design, subjective PROMs, and strong patient pre-treatment preferences introduced a credible risk of expectancy bias, leading to a high risk for D4 and overall high risk of bias.

Across the four RCTs, randomisation (D1) and missing data (D3) were generally of low risk; major limitations were concentrated in D4 and D2.

### 3.4. Synthesis of Results

#### 3.4.1. Surgery Versus Corticosteroid Injection

##### Short-Term Outcomes (≤3 Months)

Both Shahab et al., 2023 [35] and Minoğlu 2023 [34] reported faster symptomatic improvements with corticosteroid injection compared with surgery. In Shahab et al., 2023 [35], VAS pain and paraesthesia improved by approximately 19% in the steroid group versus 5–8% following CTR. The early benefit likely reflects the anti-inflammatory mechanism of corticosteroids and the delayed postoperative recovery in surgical patients.

##### Mid-Term Outcomes (6–9 Months)

At 6 months, differences in pain and functional scores narrowed substantially, with both interventions showing a progressive improvement. In Minoğlu et al., 2023 [34], although the surgical group maintained numerically superior outcomes, the confidence intervals overlapped widely, indicating clinical convergence.

##### Long-Term Outcomes (≥12 Months)

At longer follow-up, surgical decompression achieved greater and more sustained functional recovery. The DISTRICTS trial [36]—the largest and most methodologically robust RCT (*n* = 934)—reported clinical recovery (CTS-6 < 8) in 61% of surgical patients versus 45% of those initially treated with steroid injection at 18 months, yielding a relative risk (RR) = 1.36 [95% CI 1.19–1.56], an absolute risk difference (ARD) ≈ 16%, and NNT ≈ 7.

Similarly, Minoğlu et al., 2023 [34] found higher success rates—defined as the resolution of nocturnal paraesthesia—in the surgical arm (100% vs. 59.1% at 3 months; 100% vs. 31.8% at 12 months), corresponding to ARD ≈ 68% and NNT ≈ 2, although the results should be interpreted cautiously given the study’s high risk of bias.

Collectively, these findings suggest that, while steroid injection provides faster symptom relief, surgery ensures greater long-term recovery and durability of effects. Across short-, medium-, and long-term follow-up, results from individual studies are summarised in Table 2.

#### 3.4.2. Surgery Versus PENS

##### Short-Term Outcomes (≤3 Months)

In Fernández-de-las-Peñas et al., 2023 [37], PENS produced superior early outcomes. At 1 month, mean pain scores were approximately 2 points lower and BCTQ functional scores were 0.95 points better in the PENS group. These improvements reflect the neuromodulatory and analgesic action of electrical stimulation, combined with reduced invasiveness.

##### Long-Term Outcomes (12 Months)

At 12 months, pain and function outcomes were statistically and clinically equivalent between the two interventions. The absence of a long-term advantage and the single-sex design (female-only) limit generalisability.

### 3.5. Rationale for Narrative Synthesis

A formal meta-analysis was not conducted due to (1) the small number of eligible trials; (2) substantial clinical and methodological heterogeneity—differences in intervention protocols, outcome instruments, and follow-up periods; and (3) the variable risk of bias. A narrative synthesis without meta-analysis (SWiM) was therefore chosen to maintain coherence and interpretive validity without introducing artificial statistical heterogeneity. The short-term benefits of PENS compared with surgery are detailed in Table 3.

Adverse Events: Safety data were inconsistently reported and often incomplete. Across the four RCTs, only one serious adverse event (SAE) occurred—a hospitalisation for a wound infection in the surgical arm of *DISTRICTS 2025* [36]. Minor complications such as wound irritation, skin dehiscence, and delayed healing were more frequent following surgery (14%) than injection (7%). Conversely, transient sensory disturbances were reported more frequently in the injection group (72%) than in the surgery group (66%). As suggested by the trial authors, this finding may reflect the persistence or recurrence of CTS-related symptoms rather than treatment-related harm per se.

Minoğlu et al., 2023 [34] reported a single re-exploration procedure that resolved without sequelae; Fernández-de-las-Peñas et al., 2023 [37] reported no adverse events; and Shahab et al., 2023 [35] did not specify any serious events. Overall, the adverse event reporting quality was rated very low, with likely under-detection and inconsistent definitions of event severity and timing across studies.

Certainty of Evidence (GRADE): The GRADE assessment is summarised in Table 4.

- Short-term outcomes (pain and function): Certainty was low for both surgery vs. steroids and surgery vs. PENS due to the risk of bias (unblinded PROMs) and imprecision (small samples, wide confidence intervals).
- Long-term outcomes (clinical recovery): Certainty was moderate for surgery vs. steroids, supported by the large *DISTRICTS* trial and consistent directionality across studies, albeit downgraded for bias.
- Surgery vs. PENS (12 months): Certainty remained low owing to indirectness (female-only sample) and imprecision.
- Safety outcomes: Certainty was very low, downgraded for probable under-reporting, inconsistent AE definitions, and unsystematic surveillance.

Overall, evidence robustness was highest for long-term functional recovery after surgery and lowest for short-term symptom relief and safety outcomes.

Sensitivity Analyses: Sensitivity analyses excluding high-risk studies did not materially change the conclusions. For the surgery vs. steroid comparison, no low-risk trials remained to allow for quantitative pooling. For surgery vs. PENS, only one RCT was available; the findings were unchanged, confirming the stability of the narrative direction.

Clinical Implications: Overall, the evidence delineates a time-dependent treatment pattern.

- Short-term (<3 months): Corticosteroid injections and PENS offer faster symptomatic relief, likely through anti-inflammatory and neuromodulatory effects.
- Mid-term (6–9 months): Outcomes begin to converge, with both approaches showing a sustained benefit.
- Long-term (≥12 months): Surgical decompression provides the highest likelihood of durable clinical recovery, particularly in terms of functional restoration and symptom resolution.

Therefore, treatment should be individualised, integrating symptom duration, severity, patient preference, and tolerance for delayed recovery. For patients seeking rapid relief or unwilling to undergo surgery, steroids and PENS remain valid first-line options. Conversely, for those aiming at long-term resolution and functional restoration, surgical decompression appears to confer the greatest sustained benefit.

## 4. Discussion

The findings of this updated systematic review, encompassing four recent randomised controlled trials, reveal a coherent pattern in the comparative performance of surgical and conservative approaches for carpal tunnel syndrome (CTS). The synthesis highlights the temporal dynamics of the treatment response, showing that the benefits of conservative options such as corticosteroid injection and percutaneous electrical nerve stimulation (PENS) tend to be more immediate, whereas the advantages of surgery emerge gradually but persist over time.

These findings should, however, be interpreted in light of the methodological limitations of the included trials. Three of the four RCTs were judged as having a high risk of bias, mainly because of open-label designs, reliance on patient-reported outcome measures, and, in some cases, deviations from intention-to-treat analyses or treatment crossovers. As a result, the magnitude of the treatment effects, particularly for subjective outcomes such as pain and function, should be interpreted with caution.

In the short term, both corticosteroid injection and PENS provide a more rapid reduction in pain and paraesthesia compared with carpal tunnel release (CTR). This early advantage likely reflects the prompt anti-inflammatory effects of corticosteroids and the neuromodulatory impact of PENS on peripheral and central pain pathways. In contrast, surgical decompression—while addressing the mechanical source of compression—induces transient postoperative inflammation, which can delay symptom relief during the first few weeks.

Between 6 and 9 months, the differences between interventions narrow, suggesting convergence in clinical outcomes. At this stage, most patients—regardless of the treatment modality—show meaningful improvements, and early symptom advantages appear to wane. However, the long-term trajectory diverges again beyond 12 months, when surgery becomes associated with a higher probability of sustained clinical recovery, particularly in terms of functional restoration and recurrence prevention.

This long-term superiority is largely supported by the DISTRICTS trial [36], a large-scale pragmatic RCT conducted in real-world clinical conditions, which demonstrated that initial surgery increased the likelihood of clinical recovery at 18 months compared with corticosteroid injection (RR 1.36, 95% CI 1.19–1.56). Importantly, this trial provides robust, long-term evidence that the benefits of surgical decompression may extend beyond symptom relief, encompassing functional gains and reduced recurrence. Similar findings, albeit on a smaller scale, were reported by Minoğlu et al. [34], whose results pointed in the same direction but were limited by the open-label design and deviation from intention-to-treat analysis.

The inclusion of the PENS trial [37] represents another valuable addition to the evidence base. PENS showed greater pain and functional improvements at 1–3 months compared with endoscopic CTR, which may be attributed to its dual peripheral and central neuromodulatory effects and its minimally invasive nature. Nevertheless, these benefits did not persist at 12 months, and outcomes between PENS and surgery appeared similar, although the limited sample size and single-sex design preclude firm conclusions. The external validity of these results remains limited, as the study exclusively recruited women, raising concerns about sex-specific responsiveness and hormonal influences on neural excitability and pain modulation.

Compared with previous systematic reviews, including the latest Cochrane review [26], the present review provides an update based on recently published RCTs, includes follow-up data up to 18 months, and incorporates emerging interventions such as PENS. These additions refine the current evidence base, although they do not eliminate the uncertainty arising from the risk of bias and limited study numbers. 

However, the safety profile across interventions remains poorly defined. Only one serious adverse event—hospitalisation due to a wound infection—was reported, and non-serious complications were inconsistently documented. The reporting of adverse events was neither systematic nor standardised: definitions were vague, and, in some studies, adverse events were not even mentioned. Consequently, the certainty of evidence for safety outcomes remains very low, as per the GRADE criteria. This underlines the need for structured surveillance and uniform reporting frameworks in future trials, especially to balance efficacy against tolerability in real clinical contexts.

The methodological limitations of the current evidence base warrant careful consideration. Most included RCTs were open-label and relied predominantly on patient-reported outcome measures (PROMs), increasing the vulnerability to expectation and measurement bias. The lack of administering blind studies is particularly problematic in interventions where patient perception—such as postoperative recovery or immediate symptom relief—plays a substantial role in subjective reporting. Furthermore, several trials presented crossovers between groups and non-intention-to-treat (ITT) analyses, which compromise internal validity and dilute true treatment effects. Outcome instruments (e.g., BCTQ, CTS-6, VAS) and follow-up time points were also heterogeneous, precluding meta-analytic pooling.

While objective measures such as nerve conduction studies, ultrasound, or MRI could theoretically complement PROMs, their limited sensitivity and specificity for early clinical changes render them less reliable as outcome tools in CTS trials [14]. The current reliance on PROMs, although imperfect, remains a pragmatic necessity given the subjective nature of pain and paraesthesia. Nevertheless, the use of validated, standardised PROMs—ideally combined with the blinded assessment of functional tasks—could help to improve measurement consistency.

The GRADE assessment reflects these methodological weaknesses, with moderate-to-low certainty of evidence. Certainty was higher for long-term functional recovery with surgery and lower for short-term outcomes and safety due to the risk of bias, imprecision, and indirectness. The single-sex nature of the PENS trial further limits its generalisability.

From a clinical standpoint, the implications are tangible. When rapid symptomatic relief is the patient’s priority—particularly in those with mild-to-moderate CTS or high occupational demands—corticosteroid injection and PENS may represent rational initial choices, provided that patients understand that these benefits are transient. Conversely, for individuals aiming for long-lasting recovery, functional restoration, and reduced recurrence, surgical decompression should be considered the preferred option, as it directly addresses the pathophysiological compression and offers superior long-term outcomes.

Crucially, the decision-making process must remain shared and personalised, integrating patient preferences, occupational requirements, risk tolerance, and recovery expectations. The choice between conservative and surgical management should not be binary but time-contingent and goal-oriented. A stepped approach—beginning with conservative management for early relief, followed by surgery for persistent or recurrent symptoms—may provide the best balance between efficacy and invasiveness.

To advance the field, future trials should focus on (1) pragmatic, multicentre designs with rigorous ITT analysis; (2) transparent reporting of crossovers; (3) blinded outcome assessors where possible; and (4) extended follow-up of at least 24 months to capture recurrence and durability. Stratification by sex, age, and CTS severity is also essential, as the current evidence under-represents men and older adults. Furthermore, the replication of PENS findings in larger, mixed cohorts is urgently needed to validate its clinical role.

## 5. Conclusions

This updated synthesis suggests a time-dependent pattern in the comparative effectiveness of surgical and conservative treatments for carpal tunnel syndrome. Corticosteroid injections and PENS may provide faster short-term symptom relief, whereas surgical decompression appears to offer a higher probability of sustained recovery at 12–18 months. However, these findings should be interpreted with caution, as the certainty of evidence ranged from low to moderate and most included trials carried a high risk of bias. Therefore, the conclusions of this review should be considered indicative of trends rather than definitive estimates. Clinical decision-making should remain individualised and should take into account symptom severity, patient preferences, occupational needs, and recovery expectations. Further high-quality randomised trials with rigorous methodologies and longer follow-ups are needed to strengthen the evidence base.

## Figures and Tables

**Table 2:** Comparison—surgery versus corticosteroid injection. Comparison between surgical carpal tunnel release and corticosteroid injection across included RCTs. RR = Relative Risk; RD = Risk Difference; ST = Steroid Group; CH = Surgery Group; CI = Confidence Interval; mo = Months. ↑—improvement; ↓—decline; ↔—unchanged.

Study	Outcome	Total Participants	Comparison (ST/TOT ST—CH/TOT CH)	Follow-Up	RR (95% CI)	RD	Direction
Shahab et al., 2023 [35]	Numbness improvement	74	ST 7/37—CH 3/37	Short	0.43 (0.12–1.53)	−0.11	↓ Favours Steroids
Shahab et al., 2023 [35]	Pain improvement	74	ST 7/37—CH 2/37	Short	0.29 (0.06–1.29)	−0.14	↓ Favours Steroids
Minoğlu et al., 2023 [34]	Clinical success	80	ST 26/44—CH 35/36	—	1.65 (1.27–2.12)	+0.38	↑ Favours Surgery
Shahab et al., 2023 [35]	Numbness improvement	74	ST 31/37—CH 34/37	Medium	1.10 (0.92–1.30)	+0.08	↑ Favours Surgery
Shahab et al., 2023 [35]	Pain improvement	74	ST 23/37—CH 20/37	Medium	0.87 (0.59–1.28)	−0.08	↓ Favours Steroids
Minoğlu et al., 2023 [34]	Clinical success	80	ST 21/44—CH 36/36	—	2.10 (1.54–2.85)	+0.52	↑ Favours Surgery
Shahab et al., 2023 [35]	Numbness absence	74	ST 32/37—CH 34/37	Long	1.06 (0.91–1.25)	+0.05	↑ Favours Surgery
Shahab et al., 2023 [35]	Pain absence	74	ST 34/37—CH 34/37	Long	1.00 (0.87–1.14)	0.00	↔ No difference
Minoğlu et al., 2023 [34]	Clinical success	80	ST 14/44—CH 36/36	Long	3.14 (2.04–4.84)	+0.68	↑ Favours Surgery
Palmbergen et al., 2025 (DISTRICTS) [36]	Clinical recovery (CTS-6)	934	ST 180/404—CH 243/401	18 mo	1.36 (1.19–1.56)	+0.16	↑ Favours Surgery

**Table 3:** Comparison—surgery versus PENS CTS treatments. Summary of comparative outcomes between surgical decompression and percutaneous electrical nerve stimulation (PENS). NPRS = Numeric Pain Rating Scale; BCTQ = Boston Carpal Tunnel Questionnaire; CI = Confidence Interval; mo = Months. ↓—decline; ↔—unchanged.

Study	Outcome Measures	Follow-Up	Mean Difference (95% CI)	Direction	Participants (*n*)
Fernández-de-las-Peñas et al., 2023 [37]	Pain (NPRS)	1 mo	2.00 (1.10–2.90)	↓ Favours PENS	70
	Function (BCTQ)	1 mo	0.95 (0.80–1.10)	↓ Favours PENS	70
	Symptom severity (BCTQ)	1 mo	0.30 (0.07–0.53)	↓ Favours PENS	70
	Pain (NPRS)	3 mo	1.40 (0.50–2.30)	↓ Favours PENS	70
	Function (BCTQ)	3 mo	0.55 (0.30–0.80)	↓ Favours PENS	70
	Symptom severity (BCTQ)	3 mo	0.10 (−0.07–0.27)	↓ Favours PENS	70
	Pain (NPRS)	6 mo	0.50 (−0.07–1.07)	↓ Favours PENS	69
	Function (BCTQ)	6 mo	0.40 (0.22–0.58)	↓ Favours PENS	69
	Symptom severity (BCTQ)	6 mo	0.20 (0.02–0.38)	↓ Favours PENS	69
	Pain (NPRS)	12 mo	0.05 (−0.61–0.71)	↔ No difference	66
	Function (BCTQ)	12 mo	0.15 (0.00–0.30)	↔ No difference	66
	Symptom severity (BCTQ)	12 mo	0.10 (−0.06–0.26)	↔ No difference	66

**Table 4:** Summary of findings and GRADE assessment. Summary of findings according to the GRADE framework for the comparisons between surgery, corticosteroid injection, and PENS. RR = Relative Risk; MD = Mean Difference; CI = Confidence Interval; PROMs = Patient-Reported Outcome Measures; ITT = Intention-To-Treat; SAE = Serious Adverse Event.

Comparison	Outcome Measures	Studies (*n*)	Evidence Summary	Effect (95% CI)	GRADE Certainty	Downgrading Reasons
Surgery vs. Steroids	Clinical recovery ≥ 12–18 mo	2 RCTs (1014)	Favours surgery	DISTRICTS: RR 1.36 (1.19–1.56); Minoğlu [34]: RR 3.14 (2.04–4.84)	Moderate	−1 Risk of bias (open-label PROMs, crossover, non-ITT)
	Pain ≤ 3 mo	1 RCT (74)	Favours steroids	RR 0.29 (0.06–1.29); MD 0.40 (0.17–0.63)	Low	−1 Risk of bias (open-label PROMs); −1 Imprecision (small sample, wide CIs)
	Pain 12 mo	1 RCT (74)	No difference	RR 1.00 (0.87–1.14); MD 0 (−0.13–0.13)	Low	−1 Risk of bias; −1 Imprecision
Surgery vs. PENS	Pain/function ≤ 3 mo	1 RCT (70)	Favours PENS	NPRS MD 1.40 (0.59–2.21); BCTQ MD 0.55 (0.31–0.79)	Low	−1 Risk of bias; −1 Imprecision (single RCT, female only)
	Pain/function 12 mo	1 RCT (70)	No difference	NPRS MD 0.05 (−0.61–0.71); BCTQ MD 0.15 (0–0.30)	Low	−1 Risk of bias; −1 Indirectness (female only)
All comparisons	Safety (adverse events)	2 RCTs (1004) + 2 NR	Poorly reported; 1 serious event (surgery)	—	Very low	−1 Reporting bias; −1 Imprecision (rare events); −1 Indirectness (single SAE reported)

## Data Availability

Not applicable.

## Supplementary Materials

The following supporting information can be downloaded at: https://www.mdpi.com/article/10.3390/brainsci16040399/s1.
