# Supplementary material for: Surgical Versus Conservative Management for Carpal Tunnel Syndrome: An Updated Systematic Review of Randomised Trials
Source: Brain Sci. 2026 Apr 8;16(4):399. doi: 10.3390/brainsci16040399 (PMC13114700; doi:10.3390/brainsci16040399)
Supplement: Supplementary file 1 [file brainsci-16-00399-s001.zip › brainsci-4212857-supplementary.pdf]

Supplementary File

Cochrane Central Register of Controlled Trials (CENTRAL)

- #1 mh "Carpal Tunnel Syndrome" 65
- #2 "carpal tunnel" 2268
- #3 "nerve entrapment" 181
- #4 "nerve compression syndrome" 5
- #5 "nerve compression" 491
- #6 "entrapment neuropathy" 92
- #7 "entrapment neuropathies" 31
- #8 "carpal" 2483
- #9 #3 OR #4 OR #5 OR #6 OR #7 686
- #10 #9 AND #8 203
- #11 #1 OR #2 OR #10 with Publication Year from 2022 to 2025, with Cochrane Library publication date Between Nov 2022 and Jan 2025, in Trials 340

2. Cochrane Neuromuscular's Specialised Register

- #1 MeSH DESCRIPTOR "Carpal Tunnel Syndrome" with 'Neuromuscular' in Cochrane Groups 11
- #2 "carpal tunnel" with 'Neuromuscular' in Cochrane Groups 584
- #3 ("nerve entrapment" OR "nerve compression" OR (entrapment NEXT neuropath*)) AND "carpal" with 'Neuromuscular' in Cochrane Groups 47
- #4 #1 OR #2 OR #3 with Cochrane Library publication date Between Nov 2022 and Jan 2025 with 'Neuromuscular' in Cochrane Groups 4

Medline

Ovid MEDLINE(R) ALL <1946 to February 07, 2025>

- (clinical trial.pt. or exp clinical trial/ or (clin$ adj25 trial$).ti,ab. or ((singl$ or doubl$ or tripl$ or trebl$) adj25 (blind$ or mask$)).ti,ab. or placebos/ or placebo$.ti,ab. or random$.ti,ab. or research design/) not (exp animals/ not humans.sh.) 2414584
- ((Randomized Controlled Trial or Controlled Clinical Trial).pt. or (Randomi?ed or Placebo or Randomly or Trial or Groups).ab. or Drug Therapy.fs.) not (exp Animals/ not Humans.sh.) 5454080
- (comparative study/ or exp evaluation studies/ or follow up studies/ or prospective studies/ or (control$ or prospectiv$ or volunteer $).ti,ab.) not (exp animals/ not humans.sh.) 6588390
- 1 or 2 or 3 10325011
- Carpal Tunnel Syndrome.mp. or Carpal Tunnel Syndrome/ or (carp$ tunn$ or tunn$ syndrom$).mp. or (nerve entrapment or nerve compression or entrapment neuropath$).mp. 29880
- (epineurotomy or reconstruct$ or release).mp. or SURGERY/ or surgery.mp. or SURGICAL PROCEDURES, OPERATIVE/ or surgical.mp. or (splint or splints or splinting).mp. or exp Anti-Inflammatory Agents, Non-Steroidal/ or non-steroidal anti-inflammatory.mp. or NSAID$.mp. or ((corticosteroid$ or steroid$) and injection$).mp. or diuretic$.mp. or exp DIURETICS/ 5002908
- 4 and 5 and 6 5214
- limit 7 to yr="2023 -Current" 490

Embase

Embase <1974 to 2025 Week 05>

- Randomized controlled trial/ or Controlled clinical study/ or randomization/ or intermethod comparison/ or double blind procedure/ or human experiment/ or (random$ or placebo or (open adj label) or ((double or single or doubly or singly) adj (blind or blinded or blindly)) or parallel

group$1 or crossover or cross over or ((assign$ or match or matched or allocation) adj5 (alternate or group$1 or intervention$1 or patient$1 or subject$1 or participant$1)) or assigned or allocated or (controlled adj7 (study or design or trial)) or volunteer or volunteers).ti,ab. or (compare or compared or comparison or trial).ti. or ((evaluated or evaluate or evaluating or assessed or assess) and (compare or compared or comparing or comparison)).ab. 6821560

- (random$ adj sampl$ adj7 ("cross section$" or questionnaire$1 or survey$ or database$1)).ti,ab. not (comparative study/ or controlled study/ or randomi?ed controlled.ti,ab. or randomly assigned.ti,ab.) 10197
- Cross-sectional study/ not (randomized controlled trial/ or controlled clinical study/ or controlled study/ or randomi?ed controlled.ti,ab. or control group$1.ti,ab.) 422721
- (((case adj control$) and random$) not randomi?ed controlled).ti,ab. 23117
- (Systematic review not (trial or study)).ti. 311043
- (nonrandom$ not random$).ti,ab. 19862
- ("Random field$" or (random cluster adj3 sampl$)).ti,ab. 4814
- (review.ab. and review.pt.) not trial.ti. 1244091
- "we searched".ab. and (review.ti. or review.pt.) 56209
- ("update review" or (databases adj4 searched)).ab. 73681
- (rat or rats or mouse or mice or swine or porcine or murine or sheep or lambs or pigs or piglets or rabbit or rabbits or cat or cats or dog or dogs or cattle or bovine or monkey or monkeys or trout or marmoset$1).ti. and animal experiment/ 1274685
- Animal experiment/ not (human experiment/ or human/) 2683157
- 2 or 3 or 4 or 5 or 6 or 7 or 8 or 9 or 10 or 11 or 12 4687705
- Clinical Trial/ or Multicenter Study/ or Controlled Study/ or Crossover Procedure/ or Single Blind Procedure/ or Major Clinical Study/ or PLACEBO/ or Meta Analysis/ or phase 2 clinical trial/ or phase 3 clinical trial/ or phase 4 clinical trial/ or ((clin$ adj25 trial$) or ((singl$ or doubl$ or tripl$

or trebl$) adj25 (blind$ or mask$)) or placebo$ or control$ or (meta?analys$ or systematic review$) or (cross?over or factorial or sham? or dummy) or ABAB design$).tw. 17665141

- 1 or 14 19454808
- 15 not 13 16468850
- Carpal Tunnel Syndrome/ or (carpal tunnel syndrome or carp$ tunn$ or tunn$ syndrom$ or nerve entrapment or nerve compression or entrapment neuropath$).mp. 41255
- carpal tunnel release/ or epineurotomy/ or surgical approach/ or surgical technique/ or exp Nonsteroid Antiinflammatory Agent/ or exp Diuretic Agent/ or (epineurotomy or surgery or surgical or operation or reconstruct$ or splint or splints or splinting or non-steroid$ antiinflammatory or NSAID$ or ((corticosteroid$ or steroid$) and injection$) or diuretic$).mp. 7369481
- 16 and 17 and 18 7136
- limit 19 to (conference abstracts or embase) 6432
- limit 20 to yr="2023 -Current" 782

ClinicalTrials.Gov

Advanced Search

Condition or disease: Carpal Tunnel Syndrome

Study type: Interventional Studies (Clinical Trials)

First posted on or after: 01/11/2023

79 studies found

WHO International Clinical Trials Registry Platform(ICTRP)

Advanced Search

Condition: Carpal Tunnel Syndrome

Recruitment status: All

Date of registration: between 18/11/2023 and 30/01/2025

80 records found
